# The role of bone-derived factors in bone and muscle communication

**DOI:** 10.3389/fendo.2025.1702104

**Published:** 2025-10-14

**Authors:** Guobin Li, Mingyan Qi, Shibin Liang

**Affiliations:** ^1^ College of Life Sciences, Inner Mongolia Agricultural University, Hohhot, Inner Mongolia, China; ^2^ Inner Mongolia Key Laboratory of Biomanufacturing Techenology, Inner Mongolia Agricultural University, Hohhot, Inner Mongolia, China

**Keywords:** bone, muscle, endocrine organ, bone-derived factors, osteoporosis, sarcopenia

## Abstract

The interaction between bone and muscle was traditionally considered to be mechanical. However, recent insights into the endocrine functions of these two tissues have led to an emerging concept that bone-muscle biochemical crosstalk occurs through soluble factors. In light of the identification of novel bone-derived factors in recent years, more focus has been shifted to the role of bone in this partnership. Primary factors identified include osteocalcin (Ocn), fibroblast growth factor 23 (FGF23), insulin-like growth factor 1 (IGF1), sclerostin (Sost), prostaglandin E2 (PGE_2_), fibroblast growth factor 9 (FGF9), Wnt3a, and transforming growth factor beta (TGF-β). This review aims to summarize the current knowledge regarding the influence of bone-derived factors on muscle function. A comprehensive understanding of the cellular and molecular mechanisms underlying bone-muscle communication may facilitate the identification of potential therapeutic strategies for the twin diseases of osteoporosis and sarcopenia.

## 1 Introduction

Osteoporosis and sarcopenia, which are characterized by the deterioration of bone strength and muscle function, respectively, are frequently regarded as inevitable consequences of the aging process. These conditions have emerged as significant public health concerns, affecting over 1.7 billion people worldwide (1). Bone loss is often concomitant with a decline in muscle mass and function, suggesting that osteoporosis and sarcopenia develop in tandem, thereby increasing the risk of bone fractures. Bone and skeletal muscle, two major components of the musculoskeletal system, are intimately and mechanically connected. Bone serves as an attachment site for skeletal muscle, while muscle exerts force on the bone to facilitate movement (2). This mechanical linkage is essential not only for normal development and locomotion but also plays a pivotal role in the alterations of both tissues resulting from disease and aging (3). Furthermore, a growing body of evidence supports the notion that bone and muscle function as endocrine organs, affecting various other tissues and organs, including the liver, adipose tissue, kidneys, and pancreas [reviewed by Karsenty and Olson (4)]. Given the intricate relationship between bone and muscle, it is posited that the development and maintenance of these two tissues are governed by a variety of biochemical factors beyond mechanical. Currently, the growing identification of novel “myokines” has redirected attention toward the role of muscle in this interrelationship (5–7); however, the contributions of bone-derived factors and their effects on skeletal muscle should not be ignored. In this context, we aim to summarize recent findings concerning the role of bone-derived factors in the regulation of skeletal muscle function. Research on the crosstalk between bone and muscle presents a significant opportunity for identifying potential therapeutic targets for the dual conditions of osteoporosis and sarcopenia.

## 2 Bone and muscle interaction: basic and clinic evidence

Bone and muscle are originated from the same paraxial mesoderm in early fetal stage (8). An intimate association from embryogenesis, through development and growth, and into aging each other has been well-described in several excellent reviews (9–12). During embryogenesis, bone-tendon-muscle (BTM) units develop in a coordinated manner, with muscle contraction essential for bone and joint formation (13, 14). Muscle-tendon attachment sites transmit force from muscle to tendon, highlighting the interdependence of muscle and tendon development. Mesenchymal precursors condense at future bone sites, differentiating into chondrocytes or osteoblasts, guided primarily by mechanical cues (15). Once formed, bone undergoes continuous modeling during postnatal growth, followed by remodeling throughout the life cycle. This process is orchestrated by the coordinated actions of osteoclasts, osteoblasts, and osteocytes, which function to repair micro-damage and adapt to changes in mechanical stimuli. Osteoclasts are responsible for bone resorption, while osteoblasts facilitate the subsequent deposition of the mineralized matrix. Osteocytes, embedded within the mineralized bone matrix, are highly sensitive to external mechanical stimuli and adapt to changes through bone formation and resorption. Notably, osteocytes are considered the primary mechanical sensors and play a crucial signaling role. They secrete paracrine factors that regulate osteoblast and osteoclast activity, as well as endocrine signals (15, 16).

During embryogenesis, myogenesis occurs concurrently with bone development, involving the differentiation of mesodermal precursors into myoblasts, which subsequently fuse to form myotubes. Specifically, the commitment of mesodermal precursor cells to the myogenic lineage takes place within the somites of limb muscles, primarily regulated by Pax3 and Pax7. These precursors further differentiate into multinucleated syncytia, eventually maturing into muscle fibers with the involvement of external signals such as Myf5 and MyoD (17). Muscle satellite cells (SCs), a specialized subset of myogenic precursors, are located between the basal lamina and sarcolemma in resting muscles. Upon activation due to injury or growth stimuli, SCs initiate a myogenic differentiation program and integrate into existing myofibers (18, 19). In general, the increase in muscle mass and force in adults is primarily regulated by the hypertrophy of myofibers and the enhanced contractile capacity of individual myofibers, respectively. The close proximity and developmental similarities between bone and muscle suggest that overlapping signaling pathways may co-regulate the accrual of both tissues. Supporting this notion, studies have shown that MyoD-deficient mice exhibit significant abnormalities in bone development and mineralization during muscle formation (20). Conversely, bone-derived Indian hedgehog (Ihh) has been found to promote myoblast survival and myogenesis during developmental stages (21). However, it remains unclear how bone biochemically communicates with muscle during childhood and adulthood, and whether similar interactions occur in the aging process.

Osteoporosis and sarcopenia are two prevalent clinical disorders that frequently co-occur and significantly diminish the quality of life in the elderly population. Age-related sarcopenia is characterized by a systemic and progressive decline in muscle mass, strength, and function. From a biomechanical perspective, substantial evidence indicates a positive correlation between increased muscle mass and elevated bone mineral density (BMD), as well as a reduced risk of fractures (22). Furthermore, muscle strength and function have been shown to be associated with BMD and fracture risk (23). Kaji and colleagues found that higher lean body mass (LBM) is linked to increased BMD and a lower risk of fractures, particularly in postmenopausal women (24). Both muscle and bone mass can be augmented through anabolic exercise and tend to decline during periods of catabolic disuse or immobilization. Beyond mechanical interactions, bone fractures represent pathological conditions where biochemical factors significantly influence the healing process. For instance, fractures covered with muscle flaps exhibit more rapid healing compared to those covered solely with skin (25, 26). In summary, the mechanical interactions between bone and muscle, as well as the mechanisms underlying muscle-to-bone communication, have been extensively documented (10, 11, 27). However, a more comprehensive understanding of the molecular mechanisms involved in bone-to-muscle signaling remains an area of ongoing research.

## 3 Endocrine function of skeleton

Bone has long been recognized as a structural organ essential for maintaining exercise capacity, calcium homeostasis, and the hematopoietic niche. Traditionally, bone, as a target tissue, responds to hormones such as parathyroid hormone (PTH), sex steroids, and calcitonin. Emerging evidence indicates that the skeleton itself secretes at least two hormones, fibroblast growth factor 23 (FGF23) and osteocalcin, which can influence the function of distant tissues. Several comprehensive reviews have explored the endocrine role of bone in the regulation of glucose tolerance, energy expenditure, and phosphate metabolism (16, 28, 29). This underscores the notion that bone serves not only as a structural scaffold in the human body but also as a modulator of various metabolic processes. It is well-established that bone acts as a significant reservoir of circulating factors and osteogenic growth factors, which are deposited in the matrix by osteoblasts and released by osteoclasts during bone resorption. Given that osteocytes constitute approximately 90-95% of bone cells and possess extensive dendritic processes, these cells are likely the primary source of multiple circulating factors in adult bone. An initial investigation conducted by Beno and colleagues (30) revealed that molecules with a molecular weight of up to 70 kDa can readily permeate the osteocyte-lacunar-canalicular network following the injection of small dyes into the mouse tail vein. This finding suggests that canalicular fluid has unobstructed access to the circulatory system, allowing osteocyte-derived factors to potentially enter the bloodstream and exert effects on distant target cells, such as muscle cells.

## 4 Bone-derived factors and their effects on muscle

Bone serves as a key reservoir for mineral and matrix proteins and regulates skeletal muscle through the release of bone-derived factors into the bloodstream. Several of these factors include osteocalcin, FGF23, IGF1, sclerostin, PGE2, FGF9, Wnt3a, and TGF-β. We will discuss their effects on skeletal muscle, as detailed below (**Table 1**).

**Table 1:** Biological function of bone-derived factors in bone-muscle communication.

Bone-derived factors	Effect on skeletal muscle	
	*In vitro* (ref.)	*In vivo* (ref.)
IGF1	Promotes muscle cell proliferation and differentiation (31)	IGF-1 promotes protein synthesis and muscle hypertrophy via activation of PI3K/AKT pathway (3)
FGF23	FGF23 does not directly affect myotube function in skeletal muscle (32)	FGF23-mediated inhibition of insulin/IGF1 signaling is associated with muscle atrophy caused by chronic kidney disease (33)
Ocn	Enhance myoblast proliferation through PI3K/Akt/p38 MAPK and myoblast differentiation via Gprc6a-Erk1/2 signaling (34)	Increases muscle mass and function in aged mice through protein synthesis pathway (35, 36)
PGE2	Stimulates proliferation (37) and differentiation (38) of myoblasts	Increases muscle mass and strength in Cx43 transgenic mouse model (39)
Wnt-3a	Accelerates C2C12 myogenic differentiation (40)	Promotes calcium release to improve contractile force of soleus muscle (40)
Sost	Inhibits Wnt3a-mediated crosstalk between MLO-Y4 osteocytes and C2C12 myoblasts (40)	Improves muscle function in the breast cancer mouse with an anti-sclerostin antibody (41)
TGF-β	TGFβ1 induces myofibroblast differentiation and collagen deposition (42)	Excess TGF-β promotes muscle weakness related to breast cancer metastasis (43)
FGF9	Inhibits myogenic differentiation of C2C12 and human muscle cells (1)	Serves as a potential diagnostic biomarker for sarcopenia (44)

### 4.1 Osteocalcin

Osteocalcin (Ocn) is an osteoblast-specific secreted protein, and therefore its serum concentration serves as a biochemical marker for bone formation (45, 46). This small peptide is post-translationally modified on three specific glutamate residues by the vitamin K-dependent γ-glutamyl carboxylase (GGCX). GGCX catalyzes the conversion of glutamate acid into γ-carboxyglutamic acid (Gla-Ocn), which has a high affinity for bone matrix. The acidic environment generated during bone resorption promotes decarboxylation of Gla-Ocn deposited in the bone matrix to uncarboxylated osteocalcin (Glu-Ocn) and thereby increases its bioavailability. There are three factors that affect Glu-Ocn bioavailability, including leptin, glucocorticoids and delta-like 1 homolog (DLK1) (45). Thus, two forms of osteocalcin exist in the circulation, Gla-Ocn and Glu-Ocn. Only the latter form acts as a hormone (47). Glu-Ocn can signal to muscle cells via its unique known receptor: Gprotein-coupled receptor 6a (Gprc6a). A primary effect of Glu-Ocn on muscle is to increase insulin sensibility (48) and energy expenditure (49). For example, Ocn^−/−^ mice has more visceral fat without be obese than the control littermates. During exercise, Glu-Ocn signaling is conducive to the increase in muscle function. In this regard, Mera and colleagues (35) have shown, through its injections in wild-type mice and the analysis of female mice lacking Gprc6a specifically in myofibers, that bone-derived hormone Glu-Ocn enhances exercise capacity because it favors the catabolism of glucose and fatty acids in myofibers to generate ATP. Interestingly, Glu-Ocn signaling also promotes the expression and release of interleukin-6 (IL-6), a myokine whose circulating level increases during exercise. In turn, IL-6 acts in a feed-forward loop to stimulate bone resorption as well as bioactive Glu-Ocn production and secretion (50). It is worth noting that as muscle function increases, Glu-Ocn signaling also facilitates a gain in muscle mass (36). In contrast, the mice lacking tyrosine phosphatase (Esp^-/-^), an enzyme that inhibits osteocalcin function, showed an increase in muscle mass. Interestingly, muscle mass, maxmum contraction force, grip strength, fiber numbers and myosin heavy chain isoforms are altered in mice with targeted deletion of connexin 43 in osteoblast/osteocytes (51). Importantly, the Ocn circulation levels are reduced in these connexin 43 mutant mice but there is unchanged for the insulin level or glucose homeostasis. Injections of glu-OC into the conditional knockout mice rescue some of the abnormal muscle phenotypes, including body weight, cross-sectional area (CSA) and grip strength. It at least in part implied that osteocalcin is necessary to maintain muscle mass and function. An *in vitro* study revealed that glu-OC is a potent promoter of cell proliferation via PI3K/Akt/p38 MAPK pathways and myogenic differentiation via GPRC6A-ERK1/2 signaling (34). Taken together, these novel exciting data suggest that osteocalcin, especially for its active form (glu-OC), play an important role in the bone and muscle communication.

### 4.2 FGF-23

Fibroblast growth factor 23 (FGF-23), an endocrine factor produced by osteocytes, is crucial for regulating phosphate homeostasis in the kidney (52). Its expression in osteocytes is regulated by Phosphate Regulating Neutral Endopeptidase on Chromosome X (PHEX) and Dentin Matrix Protein 1(DMP1), which are promotor of bone mineralization. In the absence of either PHEX or DMP1, high levels of FGF23 promote phosphate excretion by the kidney, leading to osteomalacia and rickets. Interestingly, several lines of evidence showed that maintaining normal phosphate level may attenuate age-related muscle disorders, although the direct effects of phosphate and FGF23 on muscle have not yet dissociated (53, 54). In *DMP1^-/-^* mice, a model of autosomal recessive hypophosphatemic rickets, EDL and soleus muscles function was reduced but cardiac force production was not affected (3, 55). The deficiency of FGF23 or α-Klotho is correlated with muscle wasting, as demonstrated convincingly in animal models (56). These findings suggest that FGF23 appears to work indirectly or in synergy with the cofactors. Therefore, the use of the anti-α-Klotho neutralizing antibody to block FGF23 signaling may be an alternative strategy to maintain muscle mass without affecting renal function. Central to the role of FGF23 in bone and muscle communication, a recent report indicated that the muscle metabolite β-aminoisobutyric acid (L-BAIBA) promotes FGF23 production in osteocytes. However, this effect is lost in aged mice due to the downregulation of its receptor MRGPRD in bone (53). Interestingly, intermittent treadmill exercise favors renal phosphate excretion, which is consistent with previous findings of high levels of FGF23 with exercise are correlated with reduced phosphate (57). Overall, these data suggest that FGF23 appears to protect muscle function from increased phosphate under pathological conditions, which may be impaired during aging due to the low expression of MRGPRD and lack of BAIBA responsiveness in osteocytes.

### 4.3 IGF-1

Insulin-like growth factor1 (IGF1), produced by osteoblasts and osteocytes, is an important regulator of skeletal muscle mass by promoting both proliferation and differentiation of myoblast during muscle development. For example, adult muscle has evident and dramatic hypertrophic effect via activation of IGF-1/Akt signaling pathway (58). The hypertrophic muscle had increased absolute force but no change for specific force compared to control mice (59). In a mouse model of conditional knockout of IGF1 in osteocytes, the IGF-1 mRNA expression level was decreased in bone and muscle although no difference in muscle fiber number, size and morphology was observed (60). Similarly, bone morphogenetic protein2 (BMP2) signaling was also shown to promote adult muscle mass. However, BMP2 signaling-induced muscle hypertrophy showed an increase in absolute muscle force and unchanged or even slightly reduced specific muscle force compared to control mice (61).

### 4.4 TGF-β superfamily

Bone is a large reservoir for transforming growth factor (TGF-β), which are deposited into the mineralized bone matrix by osteoblast. The TGF-β superfamily is composed of a plethora of ligands with different selectivity for specific receptor subtypes. The TGF-β signaling is an orchestrator of diverse biological process related to proliferation, differentiation, morphogenesis, tissue homeostasis and regeneration (8). Then, subsets of TGF-β ligands family transduce the signal through downstream effectors Smad transcription factors. Activin, certain growth and difference factors (GDF) such as myostatin, GDF8, GDF11 and TGF-β can stimulate activity of smad2 and smad3. Notably, TGF-β and its family member (myostatin and activin) can lead to muscle atrophy or reduced function. Both TGF-β and activin are released into circulation from bone matrix by osteoclasts-mediated bone resorption, but their effect on muscle is different. Activin causes strongly muscle weakness with reduced muscle mass and force production using an adenovirus vector in mice. On the contrary, mice treated with TGF-β showed reduced raw and specific force production but unchanged in muscle mass (62). Waning and colleagues showed that bone-derived TGF-β is the cause of skeletal muscle weakness in the setting of osteolytic cancer through increased muscle oxidative stress and calcium mishandling (43). In contrast to the negative effects of myostatin, activin and TGF-β signaling in muscles, BMP-2 signaling leads to muscle hypertrophy as like IGF-1 (31). However, the contribution of TGF-β and BMP-2 signaling in muscle to protein balance remains to be determined.

### 4.5 Regulators of Wnt/β-catenin pathway

Wnt/β-catenin signaling is a critical pathway that affects both bone and muscle health. The osteocyte appears to transmit mechanical signals to cells on the bone surface through the wnt/β-catenin pathway. Wnt proteins are involved in embryonic muscle development and muscle growth in response to overload (57). Wnt3a was found to enhance the mRNA expression of muscle regulatory factors (MRFs)-MyoD and Myogenin, thus promote differentiation of C2C12 myoblast. Sclerostin (Sost) secreted by mature osteocytes is one of inhibitors of Wnt/β-catenin pathway. An *in vitro* study by Huang et al. (40) showed that sost inhibits the effect of Wnt 3a on the C2C12 differentiation, suggesting a potential negative role of sost in myogenic differentiation. Yet, osteocytes are mechanosensory cells that coordinate adaptive responses of the skeleton to mechanical loading although the mechanism of bone mechanosensation is not fully clear (16). Several lines of evidence showed that the expression of sost increases in the absence of load (63, 64). Thus, sost is considered to be responsible for disuse-induced osteoporosis. Irisin, an exercise hormone, is secreted by the skeletal muscle during exercise. Colaianni et al. (65) found that recombinant irisin can maintain sost expression in long bone of unloaded mice at control level. In addition, the expected increase in Rankl/OPG ratio was blunted by irisin that attenuated the disuse-induced OPG reduction. The modulation of OPG and sost by irisin could be linked, because Rankl/OPG balance is also controlled by sost (66). These findings imply that the myokine irisin modulates the sost expression, and thereby promotes osteoclast formation and activity.

Although the role of sost in bone-muscle crosstalk has been hypothesized, but its pharmacological regulation of muscle tissue is not fully clear. Romosozumab is a humanized monoclonal antibody against sost that was FDA-approved for the treatment of osteoporosis. A cross-sectional clinical study of community-dwelling elderly Korean population found that participants with sarcopenia, low muscle mass and strength have significantly lower serum sost levels (67). In addition, clinical data showed that romosozumab effectively increases BMD of lumbar spine and total hip by 13.7% and 6.2 from the baseline, respectively (68). Unfortunately, the major positive impacts of romosozumab on muscle tissue or its effectiveness in the treatment of sarcopenia are not expected.

### 4.6 PGE_2_

Prostaglandin E_2_ (PGE_2_), signaling molecule derived from arachidonic acid by cyclooxygenase (69), is related with multiple physiological process including inflammation, muscle regeneration and cancer development (38). Brotto’s lab previously demonstrated that osteocyte-derived PGE_2_ enhanced C2C12 myoblast proliferation (37) and differentiation (38), suggesting PGE_2_ is essential for skeletal muscle myogenesis. Recently, a study by Palla et al. (70) uncovered PGE_2_ signaling rejuvenates age-related decline in muscle mass and strength. Elevated 15-PGDH level in aged muscle, a PGE_2_-degrading enzyme, leads to muscle atrophy and decreased muscle strength. A physiological restoration of PGE_2_ level after 15-PGDH inhibition effectively augments mitochondrial function, thereby increasing muscle mass and strength. In addition, our group has generated two transgenic mouse models to study the distinct roles of Cx43-based hemichannels and gap junctions in bone-muscle crosstalk. Blockage of these two channels in osteocytes reduces fast-twitch muscle mass and PGE_2_ release in circulation. Interestingly, the injection of PGE_2_ to the transgenic mice partially rescues the muscle phenotypes (39). In aged transgenic mouse model, impairment of Cx43 hemichannels results in reduced PGE_2_ level in osteocytes, thereby activating TGFβ/smad2/3 pathway to promote collagen deposition in aged muscle (71). In addition to osteocytes, osteoblasts can also produce PGE_2_ and promote bone resorption, which is partially driven by activation of muscle-derived IL-6. Mechanistically, IL-6 appears to stimulate bone resorption through osteoblast-derived PGE_2_-dependent osteoclast activation (72). Therefore, PGE_2_ plays an important role in bone turnover and myogenic differentiation of myoblasts with unknown mechanisms, making it potentially essential for muscle myogenesis. These findings imply bone-derived PGE_2_ is a powerful factor in the regulation of muscle mass and strength.

### 4.7 FGF9

Fibroblast growth factor 9 (FGF9), a member of the FGF family, are mainly expressed in different cell types of skeleton. A previous study by McCormick et al. (73) showed that FGF9 was moderately expressed in primary osteocytes isolated from young mouse femurs and osteoid osteocytes. Moreover, *in vitro* experiments using the newly established osteoblast-like cell lines (OmGFP66 and OmGPF10) found that FGF23 level was significantly increased when the differentiation of osteoblasts into osteocytes. Treatment of OmGFP66 with FGF9 upregulates FGF23 mRNA expression and increases FGF23 protein secretion (74). Using C2C12 myoblast cell lines and human cells, Huang et al. (1) observed the inhibitory effects of FGF9 on myogenic differentiation, reducing the expression of myogenic factors *MyoG* and *Mhc.* Thus, FGF9 might function as a regulatory factor from bone cells to affect muscle growth, however, additional *in vivo* evidence will be required to support the concept.

## 5 Conclusions and future prospects

**Figure 1:**
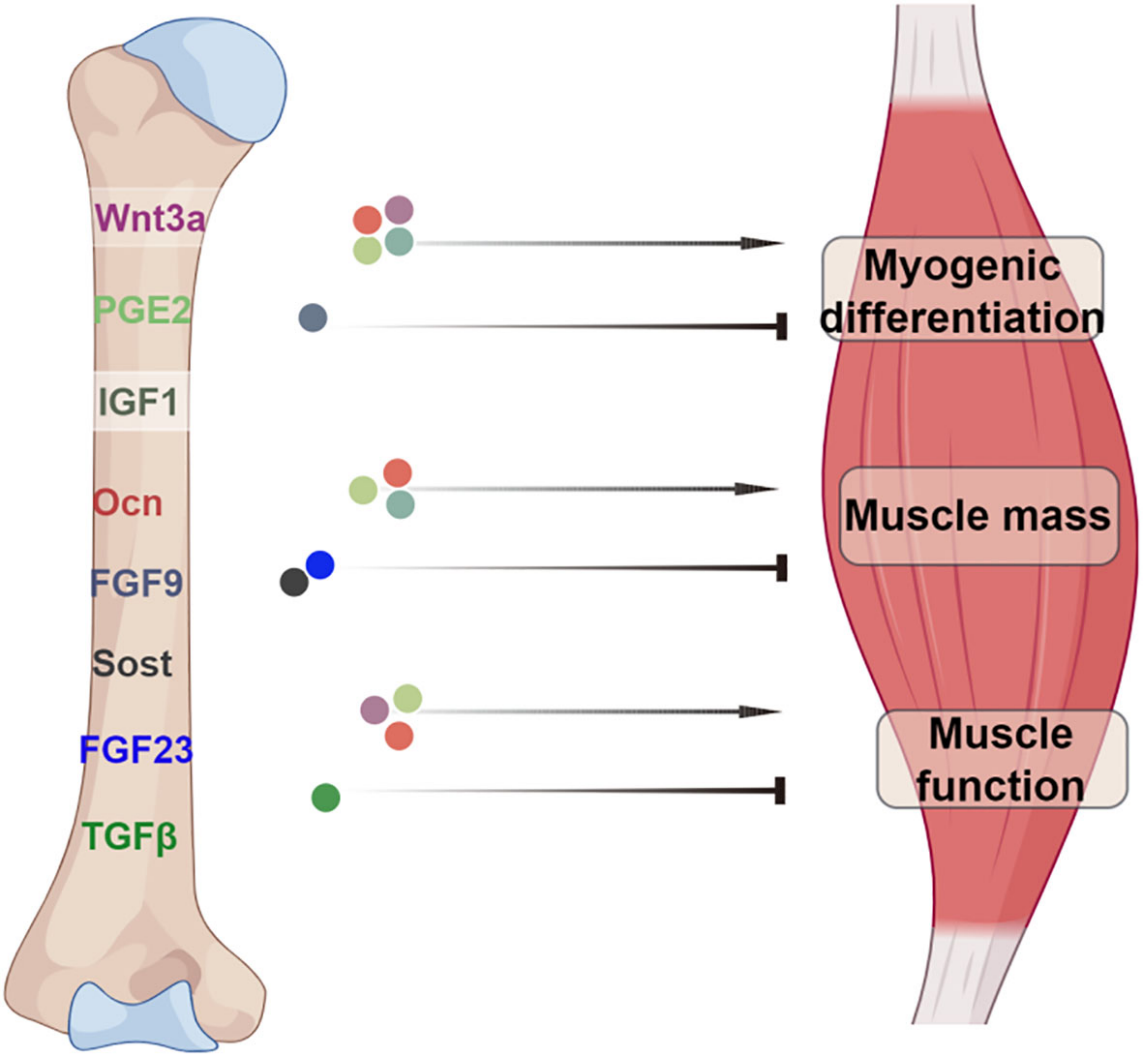
A model illustrating the role of bone-derived factors in bone-muscle axis. The bone factors Ocn, PGE_2_, and IGF1 not only have positive effects on myogenic differentiation but also on muscle mass. Wnt3a, PGE_2_ and Ocn have positive role in the regulation of muscle function. In contrast, the factors sost, FGF23, FGF9 and TGFβ exert negative effects on muscle physiology. Image created with Figdraw.com, with permission.

Evidence from numerous findings has revealed that bone and muscle are tightly coupled in both development and function. The endocrine role of bone and its interactions in bone-muscle crosstalk has recently gained great attention (75–77). Consequently, investigating the pathophysiological roles of bone-derived factors has emerged as a compelling area of research. In this concise review, we provide an overview of the current understanding of bone-muscle crosstalk and the role of bone-derived factors in regulating skeletal muscle function (**Figure 1**). However, due to the multitude of factors that similarly both affect bone and muscle, distinguishing bone-specific effects from local paracrine actions or influences originating from other organs presents a challenge. The identification of additional bone-derived factors is anticipated to offer promising targets for diagnostic biomarkers and the development of therapeutic interventions.

Age-related sarcopenia leads to impaired balance, diminished endurance, and decreased mobility, which increase the susceptibility of elderly individuals to fractures due to concurrent osteoporosis. The traditional approaches to improve bone mass and quality primarily focused on high-impact loading through gravitational forces to induce bone deformation, which is essential for bone formation. Therefore, understanding the molecular basis of bone-muscle crosstalk is crucial for developing effective therapies for the twin diseases of osteoporosis and sarcopenia, especially for the elderly who cannot bear vigorous training intervention. It is worth noting that these two diseases often exhibit sex-specific prevalence and progression in clinical practice. Thus, the effect of sex hormones on the expression of aforementioned endocrine factors should not be ignored. For example, irisin deficiency can protect osteocytes against low-calcium diet-induced bone resorption in female mice, but not male (78). Recently, Kaji’s group reported that androgen deficiency, rather than estrogen, reduces irisin expression in the muscle of mice (79). These findings suggest the sex-specific differences of irisin in bone resorption and bone-muscle communication. Pharmacological or nutritional interventions are also necessary to modify biochemical factors and signaling pathways, optimizing training strategies to reduce mechanical impacts from frailty. A key question is whether we can integrate treatments for muscle and bone diseases rather than addressing them separately. Ongoing research should aim to discover new bone-specific factors and encourage collaboration between basic, clinical, and translational studies.

## Glossary

Ocn: Osteocalcin
FGF23: fibroblast growth factor 23
IGF1: Insulin-like Growth Factor1
SOST: sclerostin
PGE2: prostaglandin E2
FGF9: fibroblast growth factor 9
TGFβ: transforming growth factor β
Pax3: paired box 3
DMP1: dentin Matrix Protein 1
Myf5: *Myogenic Factor 5*
Myod: *Myogenic regulatory factor*
SCs: satellite cells
Ihh: Indian hedgehog
BMD: bone mineral density
LBM: lean body mass
PTH: parathyroid hormone (PTH)
MAPK: mitogen-activated protein kinase
GPRC6a: G-Protein Coupled Receptor 6a
glu-Ocn: uncarboxylated osteocalcin
gla-Ocn: γ-carboxylated osteocalcin
CSA: cross-sectional area
PHEX: Phosphate-regulating neutral Endopeptidase X-linked
BMP2: bone morphogenetic protein 2
EDL: Extensor digitorum longus
GDF8: Growth and differentiation factor 8
GDF11: Growth and differentiation factor 11
15-PGDH: 15-Hydroxyprostaglandin Dehydrogenase
L-BAIBA: β-aminoisobutyric acid
MRGPRD: Mas-related G protein-coupled receptor type D
GGCX: γ-glutamyl carboxylase
Sost: Sclerostin
Rankl: Receptor activator of nuclear factor kappa b ligand
OPG: Osteoprotegerin
IL-6: interleukin6
